# Factors influencing willingness to pay for accident risk reduction among personal car drivers in Thailand

**DOI:** 10.1371/journal.pone.0260666

**Published:** 2021-11-29

**Authors:** Sajjakaj Jomnonkwao, Panuwat Wisutwattanasak, Vatanavongs Ratanavaraha

**Affiliations:** School of Transportation Engineering, Institute of Engineering, Suranaree University of Technology, Nakhon Ratchasima, Thailand; Al Mansour University College-Baghdad-Iraq, IRAQ

## Abstract

Thailand ranks near the top for the road accident fatality rate worldwide, and more and more vehicles are being registered in Thailand every year. Obtaining the opinions of road commuters may help us reduce road accidents in Thailand. This study seeks to understand damage value in road accidents for personal car drivers in Thailand, using the willingness to pay approach and establishing factors affecting willingness to pay with the theory of planned behavior (TPB). This study obtained data using questionnaires in face-to-face interviews with 1,650 personal cars drivers in Thailand. The average willingness to pay (WTP) for 50% fatality or injury reduction was 23.00 baht/person/50 km trip (US $0.74/person/50 km trip). We obtained the value of statistical life (VSL), assessing this to fall between US $815,385 and US $872,942, and the value of statistical injury (VSI), between US $150,059 and US $160,652. Overall, national damage was assessed at US $4,701,981,170 annually. According to the analysis of factors affecting WTP, TPB comprises four factors, namely, driver attitude, subjective norm, perceived behavioral control, and behavioral intention. Analysis using structural equation modeling (SEM) found all mentioned factors were relevant and positively influenced personal car drivers’ WTP in Thailand, with a statistical significance at a 99% confidence interval (*p* < 0.01). This study can develop recommendations for relevant organizations to analyze the results as part of considerations regarding budget allocation and developments on road safety policy due to driver attitude as important as environmental factors or any other factors.

## 1. Introduction

### 1.1 Background

Road accidents are drawing significant research attention. Currently, 93% of world traffic fatalities occur in low- and middle-income countries, and 60% of registered vehicles worldwide are found in these countries [1]. Recreational Vehicle-vehicle, vehicle-pedestrian, or vehicle-animal accidents [2] have significant effects, both direct ones, including injuries, medical bills, property damage, and indirect effects, including productivity loss, income loss, and mental effects on the person in the accident and others. Increasing numbers of accidents will affect the overall image of the country’s economy and society. The problem of road accidents urgently requires resolution [3, 4].

Thailand is a middle-income country [5], and it ranks near the top for dangerous road accidents. Thailand has an average road accident fatality at 32.7 persons per 100,000 population, ranking first among Southeast Asian countries and eighth worldwide [1]. The problem of road accident fatality is important and requires to be improved. In addition, personal vehicles are the most commonly represented type in accidents worldwide, suggesting regular drivers cause accidents. As many as 2,785 deaths from car accidents occur yearly in Thailand and 15,133 severe injuries [6]. The increasing fatality rate is accompanied with an increasing number of car registrations. The increasing car registrations result from the continuous growth of Thailand’s population, a symptom of increased transport demand [7]. Where transport demand is growing, but public transit cannot meet the demand, people depend on personal cars. In 2020, there were 10,880,759 car registrations, an annual average of 599,158 units, a 2.7-time increase from the annual rate 10 years ago [8]. This match accident reports finding that the road accident situation in Thailand in the last 10 years was 7.13% higher than average, with 10.26% more fatalities than average. These numbers are quite worrisome [9], and they are only exacerbated by the increasing availability of transportation and road commuting. Moreover, Thailand is a center of tourism, society, and economy of Southeast Asia in general, representing road accident problems, attitudes, and driving behaviors for vehicle and road commuters in this region.

### 1.2 Willingness to pay approach and factor affecting the willingness to pay

The willingness to pay (WTP) approach indicates the maximum value that a person would consider paying to get a unit of one thing or agrees to pay for not losing that thing [10]. This study uses WTP on transport safety to evaluate the value of a statistical life (VSL) and statistical injury (VSI) in a road accident. Using the WTP approach, we can establish how human beings evaluate their road accident risk and determine how much they would agree to pay to reduce risks [3, 4]. This can be combined with the concept value of human life, which is not restricted to seeking one’s benefit but other values, such as helping others in society [11]. WTP has recently become a more commonly used approach, as determined by analyses that have produced suitable results in safety tasks.

"Road accidents" and "road crashes" term are widely used in road safety researches, this distinction was presented by Stewart and Lord [12] who stated that accidents are the event could not have reasonably been prevented, such as a sudden change in the weather or road conditions, or rock avalanche takes you off the road as you are driving [13]. In contrast, road crashes are caused by misconduct of drivers, speeding, distracted, or careless drivers and, therefore, are not accidents [12]. In WTP studies, the "road accidents" term are suitable and widely used [14–16] as WTP is the intention to reduce the risk. These are not only caused by driver’s faults, but also raised from unpredictable events as defective car equipment or sudden environmental change.

In general, the cause of accidents has three parts, namely, human, vehicle, and environmental [17–20]. Several studies indicate that the human factor is key and should emphasize safety investigations regarding accidents [17]. Human causes are responsible for over 90% of all road accidents, not only pre-driving behavior but also during driving, such as drinking alcoholic beverages, going over the speed limit, and driving while sleepy. Physical factors in general play a role, being differentiated by the individual, such as gender [21], age, attitude, and accident experience [22]. These factors produce different driving behaviors in drivers, each of which has its risks of causing road accidents, which affect the estimation of the value of individual safety being differentiated by various basic factors of each person [14, 17, 23]. This study does not focus on obtaining a list of factors causing accidents. Instead, it focuses on factors affecting WTP for accident risk reduction based on personal factors, attitudes toward safety, perceived behavioral control, and subjective norms. All of these factors are considered within the Theory of Planned Behavior (TPB) [24] to establish the extent to which attitudes, perceptions, and intentions influence how drivers see the importance of safety. Using WTP, we consider that if drivers are willing to pay more to reduce their chance of accident risk, they are more aware of effects of a road accident.

### 1.3 Theory of planned behavior and relevant literatures

**Fig 1:**
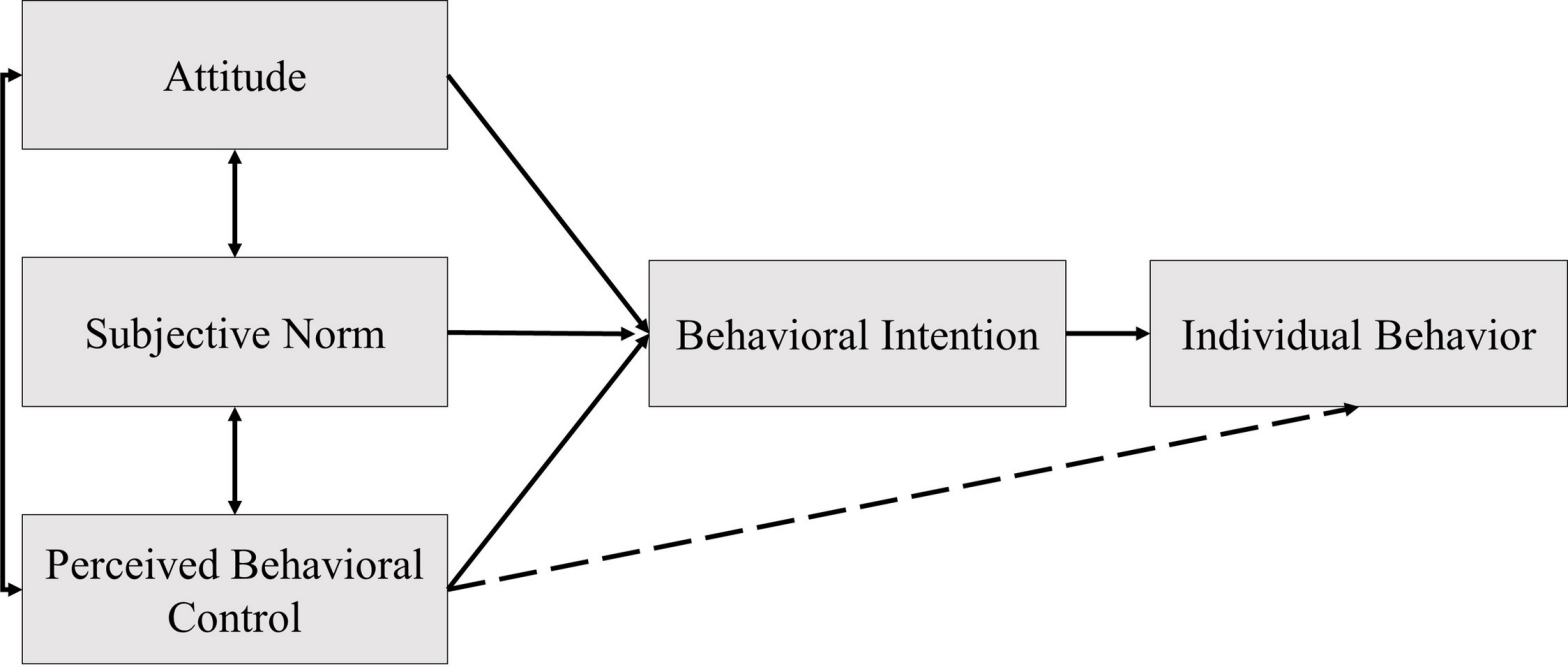
Theory of planned behavior model [25].

WTP for reducing accidents is judged according to the behavioral intention factor, which can be understood in terms of TPB, which comprises three factors that affect behavioral intention, as presented in Fig 1. 1) Attitude is the overall evaluation of any particular thing. The results of behavioral beliefs can cause one’s attitude toward behavior. If one’s evaluation of the following effect is positive, the person will have a good attitude toward the behavior. By contrast, if the evaluation is negative, the person has a negative attitude toward the behavior. 2) Perceived behavioral control is the difficult or simple responsive feeling toward behaviors caused by individual control beliefs that may support or block such behaviors, including perceived forces toward trust, which leads one to behave in a certain way or not to [24]. 3) A subjective norm represents the perception of a social trend concerning a person that leads to a certain behavior or its omission. A behavioral, subjective norm is caused by individual beliefs toward the social trend, particularly in terms of intimate friends. Normative beliefs will make a person express either one or another behavior.

Nowadays, several studies take TPB to apply its analysis along with WTP in various fields, such as the sustainability field. Obeng et al. [26] applied TPB to factors influencing WTP for rainwater source restoration in a degenerated tropical zone. Pouta and Rekola [27] took TPB to seek for factors affecting WTP to restore forest conditions in Southern Finland sustainably. In transportation and logistics, Asri and Ngah [28] analyzed factors influencing WTP for halal transportation cost with the TPB. Due to the large and growing Muslim populations in many countries, knowledge of halal transportation has come into demand.

For valuation of WTP in road accident research, one of the most widely used methods is the contingent valuation method (CVM). Several studies used CVM to collect the WTP for risk reduction, such as Ainy et al. [14], who studied the cost of road traffic injuries in Iran. Mon et al. [29] estimated the WTP and the value of fatality risk reduction for car drivers. Yang et al. [30] used WTP to estimate the VSL in China, Bhattacharya et al. [31] studied the value of mortality risk reductions in India, and Andersson [32] used WTP method to study road safety and estimate of the risk of death in Sweden. Many studies used CVM to obtain the WTP (see Table 1). This CVM involved direct questions; respondents were asked how much they were willing to pay per year for accident risk reduction or how much they were willing to pay for safety equipment per year to reduce the risk of accidents. The results of the related studies indicated many limitations in the research on the value of the accident. For example, the valuation of life or road accident is difficult for driver or road users especially when considering the value for a year as there is no criterion to measure the value clearly, driving behavior factors and road accident experiences also were overlooked, these will create gaps and driver inequality to estimate their WTP. To study WTP for road accidents based on drivers, the valuation of risk should be a concept that all drivers can valuate as clearly as possible.

**Table 1:** Previous studies on willingness to pay with contingent valuation method.

Author	Country	Method*	Willingness to pay	Analysis	Factor
Yang et al. [30]	China	CVM	risk reduction per year.	Logit model	Demographics
Mon et al. [29]	Myanmar	CVM	risk reduction per year.	SEM	Demographics
Ainy et al. [14]	Iran	CVM	risk reduction per year.	Regression	Demographics
Bhattacharya et al. [31]	India	CVM	risk reduction per year.	Probit model	Demographics
Andersson [32]	Sweden	CVM	risk reduction per year.	Probit model	Demographics
Robles-Zurita [33]	Spain	CVM	risk reduction per year.	Probit model	Demographics
Svensson and Johansson [34]	Sweden	CVM	risk reduction per year.	Regression	Demographics
Haddak [35]	France	CVM	risk reduction per year.	Tobit model	Demographics
Corso et al. [36]	USA	CVM	risk reduction per year.	Regression	Demographics
Widyastuti and Utanaka [37]	Indonesia	CVM	risk reduction per year.	Logit model	Demographics
Hoffmann et al. [38]	China	CVM	risk reduction per year.	Regression	Demographics
Alberini et al. [39]	Canada	CVM	risk reduction per year.	Regression	Demographics
Giergiczny [40]	Poland	CVM	risk reduction per year.	Regression	Demographics
Gibson et al. [41]	Thailand	CVM	risk reduction per year.	Regression	Demographics
Andersson and Lindberg [42]	Sweden	CVM	risk reduction per year.	Logit model	Demographics
This study	Thailand	CVM	risk reduction per trip kilometers.	SEM	Theory of planned behavior

*Note: CVM = contingent valuation method; SEM = structural equation modeling.

As mentioned above, the accident rate relates to increasing traffic volumes, and previous research did not consider such concerns. This study will obtain WTP for accident risk reduction of personal car drivers in Thailand based on driving distance to reduce gaps and bias. Moreover, factors affecting WTP in most previous studies are demographics. However, from a psychological perspective, intention is the best factor for predicting behavior. In this context, WTP can be predicted by intention, which is part of TPB. This study will investigate the correlation between WTP and TPB, which has never been studied in road safety research, to develop a new alternative. We will use these results as representative data of the national level. The results of this analysis can be used as a guideline for budget allocation for road accident mitigation to increase safety for road commuters and to use it as a method of study for other kinds of vehicles later.

## 2. Materials and methods

This section will explain the calculations on VSL and VSI acquired from the WTP approach. Structural equation modeling (SEM), model fit criteria, questionnaire design, and data collection.

### 2.1 Value of statistical injury and value of statistical life

This study gathered the WTP per trip for a 50% injury or fatality reduction. According to VSL and VSI, they can be measured by WTP for accident reduction, divided by decreasing numbers of injuries or fatalities [43]. In this study, we calculate the value of WTP per kilometer by using the average WTP per trip, divided by the number of kilometers per trip, as presented in Eq 1, for accident ratio per kilometer [15]. The ratio can be calculated in terms of decreasing number of annual fatalities or injuries, divided by annual vehicle kilometers traveled as presented in Eqs 2 and 3.


$$WTP\;per\;km.=\frac{WTP\;per\;trip}{Trip\;km.}$$ (1)

$$Fatality\;chance\;per\;km.=\frac{\Delta fatalities}{Annual\;VKT}$$ (2)

$$Injury\;chance\;per\;km.=\frac{\Delta injuries}{Annual\;VKT}$$ (3)

Thus, according to Eqs 1–3, we can calculate the VSL and VSI using WTP for 50% accident reduction per kilometer, divided by 50% of annual fatalities per kilometer, as presented (Eqs 4 and 5) below.


$$VSL=\frac{WTP\;per\;km.}{Fatality\;chance\;per\;km.}=\frac{WTP\;per\;Trip}{Trip\;km.}x\frac{Annual\;VKT}{\Delta fatalities}$$ (4)

$$VSI=\frac{WTP\;per\;km.}{Injury\;chance\;per\;km.}=\frac{WTP\;per\;Trip}{Trip\;km.}x\frac{Annual\;VKT}{\Delta injuries}$$ (5)

### 2.2 Structural equation modeling

SEM can be created from a theory to express the relationship between variables. Variables can be divided into two groups: exogenous or independent variables and endogenous or dependent variables [44]. This model results from a synthesis of three analytical methods including factor analysis, path analysis, and parameter estimation toward regression analysis. SEM consists of two sub-models [45], as below.

#### 2.2.1 Measurement model

A measurement model presents a relationship between the latent variable and observed variables used as indicators for each latent variable. The measurement model can be both exogenous measurement models and endogenous measurement models [46].

#### 2.2.2 Structural model

A structural model expresses the relation between exogenous latent variables and endogenous latent variables. However, the relation format is not a measurement model, but it is a path analysis from one variable to the next [47–49].

#### 2.2.3 Model fit criteria

The analysis must consider statistical values to establish whether the model can explain the following relationships to test the accuracy and model fit: the value of chi-square per degree of freedom (χ2/*df*), where initially, it should not be over 5 [50, 51]; then, the good value of root mean square error of approximation (RMSEA), which should not be specified over 0.07 [52]. Good value or Tucker–Lewis index (TLI) or non-normal fit index should be equal to or over 0.80 [53]. A suitable value of the comparative fit index (CFI) for the model is specified at/or over 0.90, and a proper value of standardized root mean square residual (SRMR) should be equal to or less than 0.08 [54, 55]. The statistical testing values can be calculated by Eqs 6–9, as follows.


$$SRMR=\sqrt{\sum_{i}\sum_{k}\frac{r_{jk}}{p^{*}}},$$ (6)

When *r*_*jk*_ is standardized residuals from a covariance matrix with j rows and k columns, and p* is the number of non-duplicated elements in the covariance matrix.


$$RMSEA=\sqrt{\frac{\chi_{T}^{2}-{df}_{T}}{{df}_{T}(N-1)}},$$ (7)

$$TLI=1-\frac{max[(\chi_{T}^{2}-{df}_{T}),0]}{max[(\chi_{T}^{2}-{df}_{T}),(\chi_{B}^{2}-{df}_{B}),0]}$$ (8)

$$CFI=\frac{\left(\frac{\chi_{B}^{2}}{{df}_{B}})-(\frac{\chi_{T}^{2}}{{df}_{T}}\right)}{(\frac{\chi_{B}^{2}}{{df}_{B}})-1}$$ (9)

Where $\chi_{T}^{2}-\chi^{2}$ values of the target model, *df*_*T*_ = *df* the target model, $\chi_{B}^{2}-\chi^{2}$ values of the baseline model, and *df*_*B*_ = *df* of the baseline model.

### 2.3 Questionnaire design

The study questionnaire (as S1 Questionaire) consists of three main sections, namely, section 1: WTP for 50% fatality or injury risk reduction by using CVM. This part is an open-ended question; respondents were asked “How much are you willing to pay to use improved roads that have 50% fatality or injury risk reduction for 50 kilometer-trip” (WTP per trip), to use this value of WTP to calculate for the VSL and VSI later. Next, section 2 concerns general information on economic and social characteristics, such as gender, age, income, education, number of people in the family, etc. This information can explain the family structure, maturity characteristics, and economic status that are the norm and differences in the sample. The final relates to the TPB obtaining the necessary information to perform SEM, while questions concerning the TPB will measure the opinion of car drivers, this section consists of four groups, including attitude, subjective norm, perceived behavioral control, and behavioral intention. The answers in this section are given on a 5-point Likert scale [56] to determine opinions on various factors phrased as statements, where 5 meant strongly agree and 1 meant strongly disagree. All questions of this questionnaire have already been passed the Index of Item-Objective Congruence test by three road safety field experts.

### 2.4 Data collection and preliminary analysis

Regarding the desirable numbers of respondents used in the analysis of SEM, in previous studies, there was a suggestion regarding suitable numbers of samples for Maximum Likelihood estimation, namely that it should be at least 15 times for the numbers of observed variables [57, 58]. In this study, we find 15 relevant variables, so there should be data from at least 225 samples before we can analyze the model properly. This study conducted its data survey using face-to-face interviews for questionnaire collection from car drivers in Thailand. Four regions of Thailand were represented (North, South, Central, and Northeast). We used random sampling from eight provinces that have the highest proportion of road accident fatalities for each region. We obtained 1,650 samples suitable for the analysis by using SEM (as S1 Dataset) and Table 2 presents the preliminary analysis.

**Table 2:** Preliminary analysis.

Category	Frequency	Percentage (%)
**Gender**		
Male	1,020	61.8
Female	630	38.2
**Education**		
Primary school	130	7.9
Lower secondary school	298	18.1
Higher secondary school/Vocational certificate	210	12.7
Diploma/high vocational certificate	126	7.6
Bachelor’s degree	802	48.6
Master’s degree	71	4.3
Doctor of philosophy	13	0.8
**Occupation**		
Student	79	4.8
Government/State enterprise officer	175	10.6
Private company	627	38.0
Self-employed	313	19.0
Farmer	139	8.4
Laborer	274	16.6
Others	43	2.6
**Accident experience**		
Never	1,405	85.2
Ever	245	14.8
**Personal income (Baht per month)**		
Less than 10,000	26	1.6
10,000 − 14,999	205	12.4
15,000 − 19,999	343	20.8
20,000 − 24,999	447	27.1
25,000–29,999	221	13.4
30,000 or higher	408	24.7
**Mean of age**	36.33 year-old	

#### Ethical approval

The ethics committee of the Suranaree University of Technology approved this study on November 13, 2020. Human research ethics application documents were submitted; then, the ethics evaluation result was that the study was low risk, it does not affect daily life, and oral informed consent is allowed.

## 3. Results

### 3.1 Descriptive statistics

Table 3 presents the descriptive statistics, including the mean, standard deviation, skewness, kurtosis, and the reliability test, obtained from the questionnaire responses to items in four groups, namely, attitude, subjective norm, perceived behavioral control, and behavioral intention (section 3 of the questionnaire). We have tested the descriptive statistic to achieve a normal distribution, following Kline [59], who indicated that a good skewness should be between −2 and 2, and kurtosis should be between −7 and 7. According to Tavakol and Dennick [60], suitable reliability should show a Cronbach’s alpha value of 0.7 or higher. The statistical values of four groups of factors were within acceptable ranges for analysis.

**Table 3:** Descriptive statistics.

Item	Description	Mean	S.D.^a^	SK^b^	KU^c^	Cronbach’s alpha
	**Attitude**					0.782
**A1**	It is useful to pay for safety on road usage because it helps to reduce the risk of accidents.	4.57	0.57	−0.96	1.14	
**A2**	To pay for safety on road usage for accident reduction makes me feel safer.	4.56	0.57	−0.87	−0.13	
**A3**	Most of my family members probably agree if I pay more for safer road usage.	4.52	0.60	−0.96	0.33	
**A4**	Most of my friends probably agree if I pay more for safer road usage.	4.51	0.62	−0.92	−0.03	
	**Subjective norm**					0.793
**S1**	Most of my family members pay for safety on road usage for accident reduction.	4.15	0.75	−0.28	−1.11	
**S2**	Most of my friends pay for safety on road usage for accident reduction.	4.18	0.75	−0.33	−1.12	
**S3**	Most people in my community pay for safety on road usage for accident reduction.	4.12	0.78	−0.22	−1.28	
	**Perceived behavioral control**					0.793
**P1**	It is my own decision to pay for safety on road usage, not by others.	4.04	0.77	−0.12	−1.17	
**P2**	Risk of accident depends on self. If I pay for safety, there will be no accident.	4.03	0.77	−0.07	−1.28	
**P3**	I can reduce accident myself by paying for safety on road usage.	4.04	0.78	−0.08	−1.33	
	**Behavioral intention**					0.732
**I1**	I will pay more for safer road usage.	4.35	0.68	−0.58	−0.71	
**I2**	I will pay for safety on road usage because I believe that it can safe my life.	4.30	0.72	−0.57	−0.69	
**I3**	I will recommend my intimates to pay for safety on road usage for accident risk reduction.	4.47	0.63	−0.85	0.15	
**I4**	I have planned to pay for safety on road usage for accident reduction.	4.51	0.61	−0.90	−0.05	

Sample size = 1,650

^a^ standard deviation

^b^ skewness

^c^ kurtosis.

In a questionnaire response where a pair of variables is very closely related, respondents likely understood them to mean the same thing. It might impede the analysis of the model and impact the correlation of observed variables that should not be over ±0.750 [61]. The correlation analysis in Table 4 indicated that no pair of variables had a higher correlation than the acceptable value, so we could take all variables to analyze the model.

**Table 4:** Correlation coefficients.

	A1	A2	A3	A4	S1	S2	S3	P1	P2	P3	I1	I2	I3	I4
**A1**	1	.292**	.210**	.130**	.074**	.080**	.063*	-.019	.054*	.044	.077**	.403**	.096**	.100**
**A2**		1	.295**	.277**	.075**	.095**	.026	.033	.108**	.059*	.162**	.137**	.261**	.311**
**A3**			1	.333**	.039	.102**	.058*	.074**	.118**	.094**	.087**	.075**	.108**	.146**
**A4**				1	.030	.106**	.048*	.076**	.088**	.115**	.030	.010	.079**	.130**
**S1**					1	.295**	.314**	-.345**	-.335**	-.347**	.001	-.114**	.070**	.080**
**S2**						1	.369**	-.313**	-.253**	-.276**	-.109**	-.107**	.004	.083**
**S3**							1	-.318**	-.336**	-.356**	-.158**	-.174**	.002	.006
**P1**								1	.571**	.581**	.321**	.184**	.207**	.049*
**P2**									1	.649**	.288**	.219**	.113**	.107**
**P3**										1	.245**	.227**	.106**	.064**
**I1**											1	.489**	.356**	.314**
**I2**												1	.287**	.244**
**I3**													1	.325**
**I4**														1

** Correlation is significant at the 0.01 level (2-tailed).

* Correlation is significant at the 0.05 level (2-tailed).

### 3.2 Estimating the VSL and VSI

We have obtained the average WTP at 23.00 baht per person per 50 km trip (US $0.74 per person per 50 km trip; exchange rate: US $1 = 31.28 baht (2020)) and a standard deviation of 16.250 baht. The estimated amount of personal car transport on the highways in Thailand in 2020 was about 7.993 x 10^10^ kilometers [62], and there were 2,785 fatalities and 15,133 injuries from car accidents in Thailand [6]. If we apply our values of WTP, annual vehicle kilometers traveled, fatalities, and injuries to Eqs 1–5, we find a VSL and VSI of car accidents, as follows.

VSL was calculated to 26,405,425 baht (US $844,163), with a 95% confidence interval of between 25,505,238 and 27,305,612 baht (US $815,385 to US $872,942).

VSI was calculated to 4,859,520 baht (US $155,355), with a 95% confidence interval of between 4,693,854 and 5,025,185 baht (US $150,059 to US $160,652).

### 3.3 Analysis of factors influencing WTP using SEM

#### 3.3.1 Structural model

**Fig 2:**
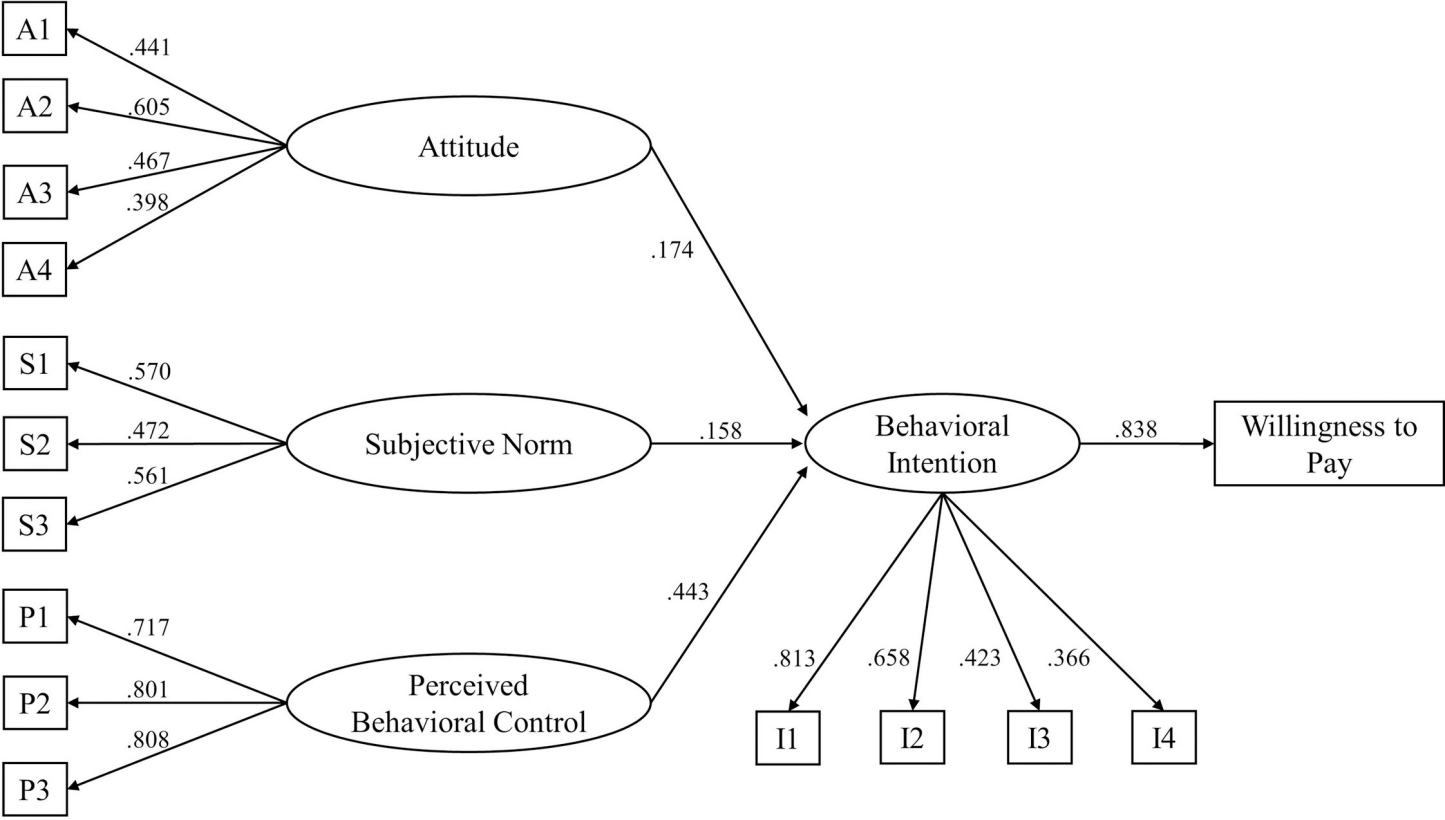
Factors influencing willingness to pay according to the structural equation modeling.

Fig 2 shows the SEM for our study on WTP for accident risk reduction using Mplus 6.12 software by Muthén & Muthén, Los Angeles, CA, USA. The analysis found that attitude, subjective norm, and perceived behavioral control influenced behavioral intention. Further, behavioral intention influenced WTP to the degree that it was significant at a 0.01 level.

To test the model fit of the SEM, the analysis found that the model had values of chi-square (χ2) = 162.841, degree of freedom (*df*) = 65, *p* < .001, (χ2/*df*) = 2.505, CFI = 0.982, TLI = 0.971, SRMR = 0.031, and RMSEA = 0.030. Comparing these statistics with acceptable values, we find that the SEM is in accordance with empirical data.

#### 3.3.2 Measurement model

The four parts of the measurement model were attitude, subjective norm, perceived behavioral control, behavioral intention, and model parameter, as shown in Table 5 and explained below.

**Table 5:** Standardized model results.

Item	Description	Standardized estimates	*t*− value	*p*− value
**Measurement model**				
	**Attitude**			
**A1**	It is useful to pay for safety on road usage because it helps to reduce the risk of accidents.	0.441	16.062	<0.001
**A2**	To pay for safety on road usage for accident reduction makes me feel safer.	0.605	18.510	<0.001
**A3**	Most of my family members probably agree if I pay more for safer road usage.	0.467	14.865	<0.001
**A4**	Most of my friends probably agree if I pay more for safer road usage.	0.398	12.500	<0.001
	**Subjective norm**			
**S1**	Most of my family members pay for safety on road usage for accident reduction.	0.570	23.756	<0.001
**S2**	Most of my friends pay for safety on road usage for accident reduction.	0.472	17.437	<0.001
**S3**	Most people in my community pay for safety on road usage for accident reduction.	0.561	22.355	<0.001
	**Perceived behavioral control**			
**P1**	It is my own decision to pay for safety on road usage, not by others.	0.717	48.154	<0.001
**P2**	Risk of accident depends on self. If I pay for safety, there will be no accident.	0.801	61.975	<0.001
**P3**	I can reduce accident myself by paying for safety on road usage.	0.808	63.324	<0.001
	**Behavioral intention**			
**I1**	I will pay more for safer road usage.	0.813	15.424	<0.001
**I2**	I will pay for safety on road usage because I believe that it can safe my life.	0.658	14.323	<0.001
**I3**	I will recommend my intimates to pay for safety on road usage for accident risk reduction.	0.423	12.207	<0.001
**I4**	I have planned to pay for safety on road usage for accident reduction.	0.366	10.711	<0.001
**Structural model**				
	Attitude → Behavioral intention	0.174	4.197	<0.001
	Subjective norm → Behavioral intention	0.158	11.943	<0.001
	Perceived behavioral control → Behavioral intention	0.443	13.474	<0.001
	Behavioral intention → Willingness to pay	0.838	14.859	<0.001

Attitude was measured by observed variables A1–A4. The analysis found that all four observed variables were components of attitude at *p* < 0.01 significance. A2 had the highest factor loading, “To pay for safety on road usage for accident reduction makes me feel safer” (*γ* = 0.605, *t* = 18.510).

Subjective norm was measured by observed variables S1–S3. The variable analysis showed that all three variables could be used as components of the subjective norm at *p* < 0.01 significance. S1 had the highest factor loading, “Most of my family members pay for safety on road usage for accident reduction” (*γ* = 0.570, *t* = 23.756).

Perceived behavioral control was measured by observed variables P1–P3. All three variables were components of perceived behavioral control at *p* < 0.01 significance. Perceived behavioral control had P3 observed variable as the highest factor loading, “I can reduce accident myself by paying for safety on road usage” (*γ* = 0808, *t* = 63.324).

Behavioral Intention was measured by observed variables I1–I4. The analysis found that all four observed variables could be used as components of behavioral intention at *p* < 0.01 significance. I1 had the highest factor loading, “I will pay more for safer road usage” (*γ* = 0.813, *t* = 15.424).

## 4. Discussion

This study obtained a VSL from road accidents of personal car drivers at approximately US $844,163, or between US $815,385 and US $872,942 in Thailand in 2020. For the VSI, it was US $155,355, or between US $150,059 and US $160,652. Comparing our results to those obtained in previous research indicated that high-income countries, calculated by gross national income (GNI) per capita, often have a higher VSL. Low- and middle-income countries have a lower VSL [5].

In a previous study, the accident value in North Cyprus, incorporating five country regions, found that the VSL was between US $380,579 and US $1,349,453. This was a high value and fluctuated due to the different physical characteristics of the regions, such that some are valleys with curves, where it is dangerous and difficult to drive, so drivers in such areas assign a higher priority to accidents than drivers living in other areas [15]. Veisten et al. [63] studied road accidents in Norway, one of the top 10 countries by per capita income, and found a VSL between US $8.81 million and US $23.05 million. This shows developed countries place a high level of importance on violent road accidents. With a significant budget allocated to road safety and enforcement of effective road laws, there are fewer accidents in developed countries than in developing ones [64, 65]. However, when each accident occurs, it causes significant damage to property [66].

On the other hand, VSL was studied in road accidents and pollution in Santiago, Chile, using the CVM and the stated preferences method, and a VSL from road accidents was around US $285,113 [67]. A study of WTP for road accident reduction in Myanmar found that the VSL was US $163,000 per person [29]. Ainy et al. [16] studied accident value in Iran, a middle-income country, found a road accident VSL of about US $22,342 per person and a VSI of US $3,138 per person, where the total value of damage from road accidents reaching US $335,003,163 per year.

Thus, higher-income countries such as Norway and Cyprus show a high value of damage from accidents. However, comparing Thailand to other low- and middle-income countries, such as Chile, Myanmar, and Iran, the value for damage from accidents in Thailand is clearly higher than its peers. It may be that drivers in Thailand consider road accidents important and agree to pay more to reduce the risk because Thailand’s rate of accident fatalities is high relative to other countries, including those of peer countries [68]. Therefore WTP for road accident reduction in each country may depend on several factors, such as socioeconomics, experience, physical characteristics of the driving area, and GNI per capita as well [15]. In summary, low- and middle-income countries tend to estimate a low value for accidents, so they cannot control and improve effective road safety. This affects the high accident ratio at over 90% for all road accidents worldwide [69]. However, in developed countries, many factors are different from those in developing countries. For example, suppose drivers have high incomes and high awareness of accident severity. In that case, they will have greater ability and intention to pay, which makes the valuation of the accident higher as well. Therefore, they can allocate a budget for proper safety management, which decreases the number of accidents. Paying for accident reduction is cheaper than having an accident, which causes income and productivity loss, as well as mental and physical damage to themselves and those close to them [3, 4].

The analysis of factors affecting WTP indicated that behavioral intention influences WTP, with a statistical significance at a 0.01 level (*γ* = 0.838, t = 14.859), which positively influences WTP. This means that if the driver has greater behavioral intention, the value of WTP will also be higher [70].

TPB, showed the factor most affecting behavioral intention was perceived behavioral control (*γ* = 0.443, *t* = 13.474). This factor is relevant to perceived behavioral self-capacity and the feeling regarding any behavior that reflects how well one considers oneself able to practice or control it [71].

The second factor that affects behavioral intention is attitude (*γ* = 0.174, *t* = 4.197). It is well known that any behavioral expression is caused by an attitude toward behavior. That is, if one has bad or negative attitudes toward a behavior, this will decrease behavioral intention. In conclusion, if a driver is not interested in road accidents, this affects WTP accordingly [72]. The obtained result showed that attitude positively influenced behavioral intention, which is consistent with previous research [70]. This means that where attitudes toward safety improve among drivers, they will agree to pay more for it.

The third factor was the subjective norm (*γ* = 0.158, *t* = 11.943), or the influence of society or intimates that affects one’s behavior. The subjective norm develops from personal belief concerning social trends, particularly in relation to one’s intimates. In other words, if family members, for instance, show a certain behavior, others may follow the same behavior, in a phenomenon called conformance. If one’s intimates have a preferred behavior regarding safety on the road for accident reduction, it will influence others in the same society or environment to have the same behavior. According to the results obtained, subjective norms have positive influences on behavioral intention, which means that drivers tend to behave in the same way as their intimates behavior. However, this part has the lowest factor of all three factors. That is, drivers still prioritize attitudes and perceived behavioral control toward road safety themselves rather than conforming to those of others [73].

## 5. Conclusions

This study collected VSL and VSI concerning road accidents among personal car drivers in Thailand using the WTP approach, including the study of factors affecting the value of an accident. The analysis was divided into two parts, consisting of the following. 1) WTP for accident risk reduction, implying a VSL between US $815,385 and US $872,942 and a VSI between US $150,059 and US $160,652, for a total national value of property damage at US $4,701,981,170 per year. 2) For factors affecting WTP, this study developed a model with the TPB using SEM. The analysis result indicated that the three factors of attitude, subjective norm, and perceived behavioral control positively influence behavioral intention. Further, behavioral intention positively influences WTP for accident risk reduction with statistical significance at a 0.01 level. Thus, TPB was associated with attitude to pay for accident risk reduction, while subjective norm related to the influence of one’s intimates, which lead to drivers’ behavior. Drivers themselves cause the relation to attitude and perceived behavioral control. That is, producing a good attitude toward drivers’ road safety allows them to develop knowledge and understanding, as they place more importance on road accidents. Following this, these drivers will pass on such behavior to others as well.

As it moves toward becoming a fully developed country, road accidents are a main issue that Thailand must address [62] to allow different purposes of traveling and transport to operate safely and efficiently. The uniqueness of this study is that we do not study the value of accidents based on basic factors or the components of accident occurrence, as in other research. However, we focus on the importance of attitudes and perspectives of drivers toward road accidents, which are human factors. The analysis results indicated that good attitudes on accident reduction, perceived behavioral control of drivers, and subjective norm of one’s intimates increased behavioral intention relevant to road safety and increased the value of an accident. Therefore, we recommend that organizations who work on road safety management take this result as a guideline for budget allocation regarding accidents, including the development of the policy of road accident reduction. Organizing training on attitudes toward road safety or adding such lessons into training programs for people who apply for car driving licenses raises awareness that drivers’ attitudes are as important to road safety as any other factor.

Among the limitations of this study is that we did not allow sociodemographics influencing the WTP of drivers, this could help relevant authorities to promote specific Road Safety Education programs [74]. Moreover, we did not include drivers under 18 years old in the analysis due to licensing laws in Thailand. Because adults are often more intellectually independent, certain factors such as attitude or subjective norm might be different among the young. A similar study on a younger age group may produce different results and capture a more representative share of the population.

## Supporting information

S1 Questionaire (DOCX)

S1 Dataset (XLSX)
