# Supplementary material for: Factors influencing willingness to pay for accident risk reduction among personal car drivers in Thailand
Source: PLoS One. 2021 Nov 29;16(11):e0260666. doi: 10.1371/journal.pone.0260666 (PMC8629291; doi:10.1371/journal.pone.0260666)
Supplement: S1 Questionaire — (DOCX) [file pone.0260666.s001.docx]

**Questionnaire: Value of Road Accident for Personal Car Drivers in Thailand**

**ID: ................Date..............**

School of Transportation Engineering, Institute of Engineering, Suranaree University of Technology

**Objective:** This questionnaire aims to obtain the value of road accidents among personal car drivers in Thailand using willingness to pay method. To estimate the value of road accident and develop model of factors affecting the value of the road accident. In order to provide guidelines and policies recommendation for involved organization.

The questionnaire has 2 pages and consists of 3 parts as follows:


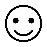

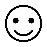

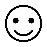

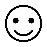

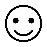

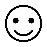

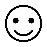

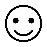

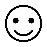

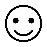

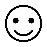

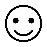

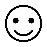


**Section 1 Estimating Willingness to Pay (Contingent Valuation Method)**

**References in your area,**

When you travel on type A roads (for 50 kilometers), it will cost about 80 baht (gasoline, deteriorate and other expenses).

how much you willing to pay more for using improved road?


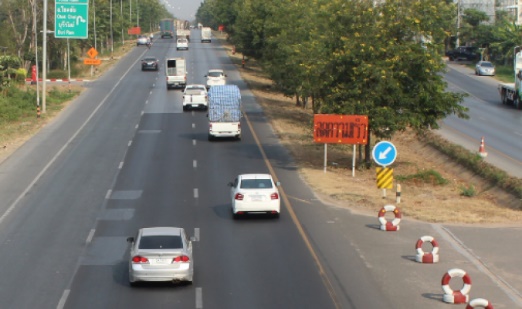


**Type A (Inter-city route)**

***Current route***

Accident rate:

32.7 persons/100,000 population


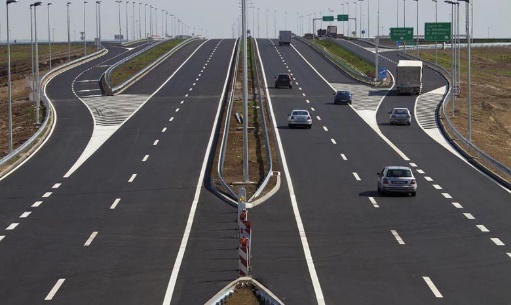


**Type B (Inter-city route)**

***Improved route***

Accident rate:

16.4 persons/100,000 population

How much you willing to pay more for 50 km. improved road (type B)?

****The maximum amount you are willing to pay =........................ baht per trip (50 km.)****

**Section 2 General information and socioeconomics**

**Description:** Please mark ✓ in front of your answer.

***(Every data is important to the analysis. Please provide this information and information is concealed.)***

**2.1) Age…………year 2.2) Gender** ⭘ 1) Male ⭘ 2) Female

**2.3) Education** ⭘ 1) Primary school ⭘ 2) Lower secondary school

⭘ 3) Higher secondary school/Vocational certificate ⭘ 4) Diploma/high vocational certificate

⭘ 5) Bachelor’s degree ⭘ 6) Master’s degree

⭘ 7) Doctor of philosophy ⭘ 8) Other

**2.4) Personal income** *(For student, put the income received from parents/part-time jobs.)***…….…………Baht/month**

**2.5) Occupation** *(choose 1 option)* ⭘ 1) Student ⭘ 2) Government/State enterprise officer ⭘ 3) Private company ⭘ 4) Self-employed ⭘ 5) Farmer ⭘ 6) Laborer ⭘ 7) Others

**2.6) Accident experience** ⭘ 1) Ever ⭘ 2) Never

**Section 3 Theory of Planned Behavior**

**Description:** Please mark ✓ in the answer box that corresponds to your opinion

| **No.** | **Question** | **Strongly agree<->Strongly disagree** | | | | |
| --- | --- | --- | --- | --- | --- | --- |
|  |  | **5** | **4** | **3** | **2** | **1** |
|  | **Attitude** |  |  |  |  |  |
| 3.1 | It is useful to pay for safety on road usage because it helps to reduce risk of accident. | 5 | 4 | 3 | 2 | 1 |
| 3.2 | To pay for safety on road usage for accident reduction makes me feel safer. | 5 | 4 | 3 | 2 | 1 |
| 3.3 | Most of my family members probably agree if I pay more for safer road usage. | 5 | 4 | 3 | 2 | 1 |
| 3.4 | Most of my friends probably agree if I pay more for safer road usage. | 5 | 4 | 3 | 2 | 1 |
|  | **Subjective norm** |  |  |  |  |  |
| 3.5 | Most of my family members pay for safety on road usage for accident reduction. | 5 | 4 | 3 | 2 | 1 |
| 3.6 | Most of my friends pay for safety on road usage for accident reduction. | 5 | 4 | 3 | 2 | 1 |
| 3.7 | Most people in my community pay for safety on road usage for accident reduction. | 5 | 4 | 3 | 2 | 1 |
|  | **Perceived Behavioral control** |  |  |  |  |  |
| 3.8 | It is my own decision to pay for safety on road usage, not by others. | 5 | 4 | 3 | 2 | 1 |
| 3.9 | Risk of accident depends on self. If I pay for safety, there will be no accident. | 5 | 4 | 3 | 2 | 1 |
| 3.10 | I can reduce accident myself by paying for safety on road usage. | 5 | 4 | 3 | 2 | 1 |
|  | **Behavioral Intention** |  |  |  |  |  |
| 3.11 | I will pay more for safer road usage. | 5 | 4 | 3 | 2 | 1 |
| 3.12 | I will pay for safety on road usage because I believe that it can safe my life. | 5 | 4 | 3 | 2 | 1 |
| 3.13 | I will recommend my intimates to pay for safety on road usage for accident risk reduction. | 5 | 4 | 3 | 2 | 1 |
| 3.14 | I will pay more for safer road usage. | 5 | 4 | 3 | 2 | 1 |
